# Suicide prevention curriculum development for health and social care students: A scoping review

**DOI:** 10.1371/journal.pone.0328776

**Published:** 2025-07-28

**Authors:** Clíodhna O’ Brien, Kerrie Gallagher, Michelle O’ Driscoll, Doireann Ní Dhálaigh, Paul Corcoran, Mette Valdersdorf Jensen, Eve Griffin

**Affiliations:** 1 National Suicide Research Foundation, Cork, Ireland; 2 School of Pharmacy, University College Cork, Cork, Ireland; 3 School of Public Health, University College Cork, Cork, Ireland; 4 Centre for Suicide Research, Odense, Denmark; 5 Department of Regional Health Research, University of Southern Denmark, Odense, Denmark; Lahore Medical and Dental College, PAKISTAN

## Abstract

**Background:**

Suicide is a widely recognised public health concern. International evidence indicates that many individuals who die by suicide have had contact with a healthcare professional in the year preceding their death. Moreover, the evidence regarding the training of healthcare professionals is concerning and points to gaps in the provision of training to adequately prepare health professionals in responding to and assisting individuals in a state of suicidal crisis. There is a recognised opportunity to ensure that all health and social care students, regardless of their discipline, receive formal suicide prevention training. Despite this imperative need, there is a notable absence of literature summarising the current state of such training across healthcare disciplines. This scoping review aimed to identify literature describing the design, development, implementation, and/or evaluation of suicide prevention training for healthcare and/or social care students in higher education settings.

**Methods:**

Following a predetermined protocol, we conducted a scoping review adhering to PRISMA guidelines for Scoping Reviews (PRISMA-ScR). The author team formulated a search strategy incorporating variations of keywords such as “*student*,” “*suicide prevention*,” and “*education*.” The search spanned six databases—PubMed, ERIC (Education Resources Information Center), CINAHL, Embase, PsycInfo (EBSCO), and Web of Science. Additionally, grey literature sources were explored, alongside forward and backward citation searches of the included articles. Two reviewers independently carried out title and abstract screening, as well as full-text screening. Data extraction from the included studies was also conducted independently by two reviewers, with any discrepancies resolved through group consensus. A narrative summary of key findings was developed.

**Results:**

In total 58 articles were included which detailed several programmes conducted mostly in the United States of America and Australia and were targeted at a variety of healthcare students. When specified, learning outcomes were associated with improving attitudes and developing knowledge. The programmes employed diverse teaching strategies, including lectures, role-playing, and patient simulations. While student evaluations generally showed improvements in knowledge, confidence, and preparedness, the evidence on the effectiveness of different instructional approaches remains inconsistent.

**Conclusion:**

By integrating comprehensive suicide prevention training into health and social care curricula, there is an opportunity to instil the knowledge, skills, and attitudes necessary to effectively address suicide risk. Further research is warranted to elucidate the most effective delivery methods and teaching modalities for suicide prevention training programmes in health and social care students, with scope for further exploration of interprofessional learning opportunities in this area. The development of internationally recognised core competencies and learning outcomes for health and social care students in this area is also critical to ensure a consistent, effective approach to suicide prevention across healthcare and social care settings.

## Introduction

Suicide is a serious public health concern and is one of the leading causes of preventable deaths globally [1]. Research conducted in the United States indicates that over 90% of individuals who died by suicide had engaged with healthcare services in the year leading up to their death, with an average of 16.7 healthcare visits during that time [2]. In Ireland, a recent psychological autopsy of 307 suicide cases revealed that 80.1% of suicide decedents had recent contact with their general practitioners (GPs), 84.7% were diagnosed with a mental disorder, 60.7% had substance abuse issues, and 30.6% faced physical health problems [3]. This underscores the diverse array of health and social care professionals who may encounter individuals exhibiting suicidal intent, presenting a crucial opportunity for intervention. These professionals may include primary care or hospital-based physicians, surgeons, addiction counsellors, general or mental health nurses, physiotherapists, pharmacists, occupational therapists, social workers, or psychotherapists.. Their roles may include conducting initial assessments, offering brief psychosocial support, facilitating referrals to mental health services, contributing to safety planning, and in some cases, providing structured therapies, and ensuring safe medication practices [4]. As gatekeepers within the healthcare system, health and social care professionals are well-positioned to coordinate timely, multidisciplinary responses. Their capacity to fulfil these responsibilities depends on adequate training, confidence, and access to suicide prevention education. Additionally, contact with primary healthcare is particularly common in the final month before death [4], emphasising the role of medical professionals in recognising those who may be at risk and implementing evidence-based suicide prevention strategies [5].

Nonetheless, research consistently shows that many healthcare professionals lack the knowledge, skills, and confidence to implement such strategies [5–8]. This gap in proficiency highlights the urgent need for comprehensive training and support systems to equip healthcare professionals with the necessary tools and confidence to address suicidal behaviours effectively.

Targeting healthcare students for suicide prevention training offers a promising avenue to ensure a consistent and comprehensive approach within the healthcare system. Furthermore, healthcare professionals’ attitudes toward suicide and prevention are shaped during their undergraduate years [9]. In the United States, recommendations from the Office of the Surgeon General advocate for incorporating suicide prevention competencies into undergraduate and graduate programmes for various healthcare professions [10]. This endorsement highlights the importance of integrating suicide prevention training into the educational pathways of future healthcare professionals, ensuring they are well-prepared to address this critical aspect of patient care [10].

Despite the critical need for such training, there is a lack of formal education on suicide prevention for healthcare students [4,11,12]. Factors contributing to this gap include discomfort with the topic, limited time in the curriculum and competing educational priorities [13,14]. Research suggests that trainee healthcare professionals may encounter patients with suicidal ideation but receive insufficient training on how to handle such situations [15]. For example, a survey of pre-doctoral psychology interns indicated that 99% had treated a person with suicidal intent, but only 50% had received formal suicide prevention training [16]. Healthcare students commonly express fear and lack of confidence in treating suicidal patients, with undergraduate medical students rating their interpersonal suicide prevention skills as poor [17]. Training healthcare students could enhance their confidence in responding to patients with suicidal intentions or behaviours in their future professional endeavours [7]. To the authors’ knowledge, there has been no prior reporting on the evidence related to a suicide prevention curriculum which incorporates all health and social care students.

The aim of this scoping review was to identify any relevant literature which documented the design, development, implementation, and/or evaluation of a suicide prevention course for undergraduate and postgraduate students of health and social care degree programmes.

## Materials and methods

This scoping review followed the PRISMA-ScR guidelines [18] (see S1 Table: PRISMA-ScR Checklist). A pre-determined protocol was established for the conduct of this review [19]. We opted for a scoping review approach due to the evolving nature of evidence on suicide prevention curricula, especially concerning health and social care students. The scoping review methodology is fitting for this study, as its primary objective is to identify studies covering various aspects such as the design, development, implementation, and/or evaluation of suicide prevention training specifically tailored for healthcare and/or social care students in higher education settings. This review implemented Arksey and O’Malley’s framework [20] for scoping reviews, employing five key stages to guide the study design: (i) formulating the research question; (ii) identifying pertinent studies; (iii) selecting studies; (iv) charting data; and (v) synthesising, summarising, and reporting the findings.

### Formulating the research question

The scoping review aims to address the following question:

“What are the existing practices in the design, development, implementation, and/or evaluation of suicide prevention training tailored for healthcare and/or social care students in higher education settings?”

#### Inclusion and exclusion criteria.

Studies eligible for inclusion in this review were those that investigated suicide prevention training provided to undergraduate or postgraduate students pursuing health and/or social care degrees within higher education settings, such as colleges and universities. Quantitative, qualitative, and mixed method studies, as well as any studies that described the development of a suicide prevention module in higher education settings were included. Any relevant grey literature was also included. However, for quality assurance and consistency, only peer-reviewed publications were included in the final review. This criterion was applied to ensure that all included sources had undergone scholarly review and met academic standards of evidence.

Given the exploratory nature of the research, no control group was required, and all studies addressing any aspect of the design, development, implementation, and/or evaluation of suicide prevention training for healthcare and/or social care students in higher education settings were included. There were no geographical limitations, but due to resource constraints, only studies published in English since January 1, 2011, were included. Exclusions were applied to studies reporting on suicide prevention training outside of the higher education curriculum for health and/or social care students (i.e., for professionals in these fields), studies where data specific to health or social care students could not be separated from other student cohorts, studies in languages other than English, and those published before January 1, 2011.

### Identifying relevant studies

#### Search strategy and information sources.

The search strategy was collaboratively developed by the author team, who have significant expertise in the area of mental health and suicide prevention research. Additional input was sought from an academic librarian affiliated with the author’s institution. The search was implemented across six databases: PubMed, PsycInfo (EBSCO), ERIC, Web of Science Core Collection, CINAHL and Embase. The search was run on the 25^th^ of July 2023 and subsequently updated on the 7^th^ of July 2024. The strategy was customised for each specific database, incorporating MeSH terms where applicable, and was restricted to titles, abstracts, and keywords in databases that supported it. The search included various iterations of the terms “*students*”, “*suicide prevention*”, and “*education*”. To ensure global coverage, the search terms were deliberately broad and included synonyms and terms relevant to both high and low-middle income settings, no geographical filters were applied. An example of the search strategy used, and the corresponding outcomes are outlined in the S2 Table: Search strategy and results. The indexes and editions available through the author’s institutional subscription to Web of Science Core Collection can be viewed in the S3 File: Indexes in Web of Science Core Collection.

In addition to the database searches, the research extended to explore pertinent grey literature sources to capture items not typically accessible through database searching. In addition to the six academic databases searched, we explored grey literature using Google Scholar, the Suicide Prevention Resource Center (SPRC), BASE and ResearchGate. These sources were reviewed to identify potentially relevant materials; however, only four grey literature documents were ultimately cited, as we did not identify other eligible peer-reviewed publications through these platforms that met the inclusion criteria. A manual search of the reference lists of the included studies was carried out to identify any potentially overlooked materials. Additionally, a forward citing search was conducted to identify articles that cited the articles identified through our initial search, further enhancing the scope of the review.

### Study selection

Following the search process, all identified citations were gathered and uploaded to the reference manager software Zotero, Version 6.0.20. Citations were subsequently imported into Rayyan [21], and any duplicates were systematically eliminated. Both title and abstract screening and full text screening were independently undertaken by two reviewers (KG and COB or MOD). Any conflicts arising from this process were discussed, and a third author consulted (EG or PC) where consensus could not be reached.

### Charting the data

Data extraction was independently conducted by three researchers (KG, MVJ, MOD). The data extracted from the relevant studies was entered into an electronic spreadsheet created in Excel (S4 Table: Data extraction template). The recorded data encompassed information including the authors’ names, country of origin, publication year, study setting, study population details, programme specifics, associated learning outcomes, implementation methods of the programme, and the methodologies employed in the respective studies. The data was checked for discrepancies by one reviewer (KG) and any conflicts arising were discussed by the review team until consensus was reached. The data extraction template is available in the S3 Table: Data extraction template.

Consistent with the scoping review research design, the methodological quality of the included articles was not reported or assessed. Scoping reviews typically prioritise breadth and inclusivity over a detailed evaluation of study quality, making this approach in line with the exploratory nature of the research.

### Collating, summarising, and reporting the results

Descriptive statistics were generated to provide a comprehensive summary of the key characteristics of the included studies. In addition, a narrative synthesis was conducted to explore and integrate the findings related to learning outcomes, teaching methodologies, and evaluation outcomes, where applicable. This approach allowed for a cohesive interpretation of the studies by identifying commonalities, and emerging patterns across the various programmes.

## Results

The initial database search was conducted on June 27, 2023, yielding 2,025 results. After adding four records from grey literature sources via Google Scholar [22–25] and removing duplicates, a total of 945 records remained for the title and abstract screening stage. No additional references that met the inclusion criteria were found via SPRC, BASE or ResearchGate. 91 articles were assessed for eligibility through full-text review, and 46 were included. An additional nine articles were identified and incorporated through forward and backward citation searches, bringing the total to 55 articles.

**Fig 1:**
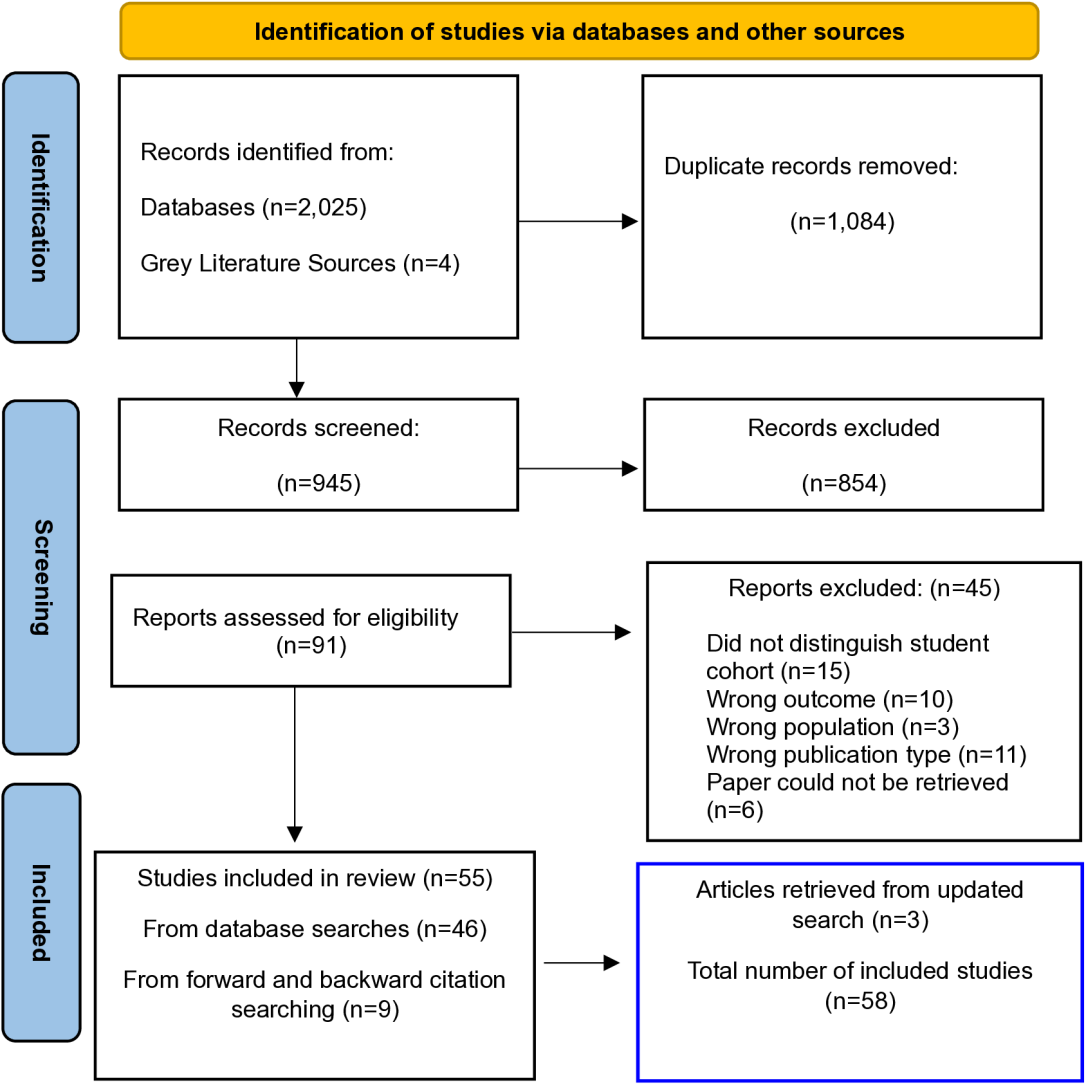
PRISMA flow diagram.

A subsequent search was re-run across all six databases on July 5, 2024, resulting in the identification of three new articles. Therefore, the final number of articles included in the review was 58. Fig 1 provides a visual overview of the flow of information throughout the distinct phases of this scoping review, detailing the process from initial search to final inclusion.

### Programme details

The included articles outlined several programmes conducted mostly in the United States of America (USA) (*n* = 32, 55%) and Australia (*n* = 7, 12%). The remaining programmes were delivered to student populations in Canada, the United Kingdom, India, Singapore, Taiwan, Belgium, France, Spain, the Netherlands, Japan, and China. Programmes were targeted at a variety of healthcare students including Social Work, Pharmacy, Medicine, Nursing, Psychology and Youth Work, with some programmes aimed at interdisciplinary teaching (*n* = 10, 17%). Most programmes were delivered to undergraduate students (*n* = 40, 69%), with the remainder delivered to postgraduates (*n* = 13, 22%) or both undergraduate and postgraduate students (*n* = 5, 9%). There was a broad mix of when the suicide prevention training was implemented ranging from first to final year students and the number of students in courses ranged from 5 to 1345 in total. Session lengths spanned from fifteen minutes to seven and a half hours. Number of sessions also varied from one session to semester long undergraduate courses containing 16 sessions [26]. Table 1 outlines the breakdown of programme details.

**Table 1:** Programme details.

Authors	Country	Setting	Degree course(s)	Stage of the degree	Student population	Students (n)	Sessions (n)	Session length (hr:mins)	Core or elective course
Almeida *et* *al*., 2017 [14]	USA	Graduate school	Social Work	Mixed	Postgraduate	22	14	02:50	Core
Afsharnejad *et al*., 2022 [61]	Australia	University	Interdisciplinary	Mixed	Undergraduate and Postgraduate	129	6	Unknown	Unknown
Bornheimer *et* *al*., 2024 [39]	USA	University	Social Work	Unknown	Postgraduate	22	2	05:00	Elective
Boukouvalas et al., 2018 [62]	Australia	University	Pharmacy	Mixed	Undergraduate and Postgraduate	252	15	Unknown	Unknown
Carpenter *et* *al*., 2023 [37]	USA	University	Pharmacy	Mixed	Postgraduate	180	1	1:15	Elective
Cates and Woolley, 2017 [5]	USA	University – clinical placement	Pharmacy	4^th^ year	Undergraduate	41	Unknown	Unknown:	Unknown
Chuop *et* *al*., 2021 [44]	USA	University	Medicine	2^nd^ year	Undergraduate	154	2	00:50	Unknown
Cramer *et al*., 2019 [40]	USA	University	Interdisciplinary	Mixed – introduced at various stages of degree course	Undergraduate and Postgraduate	32	15	Unknown	Unknown
Cramer & Long, 2018 [27]	USA	University	Interdisciplinary	Mixed – introduced at various stages of degree course	Undergraduate	20	6	Unknown	Core
Cramer *et al*., 2016 [52]	USA	University	Psychology	Mixed – introduced at various stages of degree course	Postgraduate	5	Semester long course	Unknown	Unknown
De Silva *et* *al*., 2015 [17]	Australia	University	Medicine, Paramedicine and Pharmacy	1^st^ year	Undergraduate	542	1	05:00	Core
Desai *et al*, 2018 [53]	India	University – clinical placement	Medicine	Unknown	Undergraduate	32	4	03:00	Unknown
El-Den *et* *al*., 2018 [28]	Australia	University	Pharmacy	4^th^ year	Undergraduate	163	Unknown	Unknown	Core
Goh *et al*., 2016 [29]	Singapore	University	Nursing	2^nd^ year	Undergraduate	95	2	Unknown	Core
Harshe *et* *al.*, 2022 [30]	India	University	Medicine	Unknown	Undergraduate	57	1	01:20	Core
Heyman *et al*., 2015 [31]	Scotland	University	Mental Health Nursing	2^nd^ year	Undergraduate	27	2	Unknown	Core
Hjelvik *et* *al.*, 2022 [25]	USA	University	Medicine	Mixed	Undergraduate	273	1	1:45	Unknown
Hill et al., 2024 [63]	USA	University	Psychology	Unknown	Undergraduate	Intervention group (n = 179) Control group (n = 195)	1	01:00	Unknown
Hutson and Zeno, 2021 [54]	USA	University	Nursing	Unknown	Undergraduate	Unknown	Unknown	00:20	Unknown
Jacobson *et al*., 2012 [41]	USA	University	Social Work	2^nd^ year	Postgraduate	72	1	01:30	Unknown
LeCloux, 2021 [22]	USA	University	Social Work	Unknown	Postgraduate	53	Unknown	Unknown	Core
Kerr *et al*., 2018 [32]	United Kingdom	University	Mental Health Nursing	1st year	Undergraduate	128	1	03:30	Core
Kourgiantakis *et* *al*., 2021 [24]	Canada	University	Social Work	Mixed	Postgraduate	15	1	Unknown	Unknown
Kratz et al., 2020 [23]	USA	University	Social Work	1st year	Postgraduate	58	4	Unknown	Unknown
Kullberg *et* *al*., 2020 [64]	Netherlands	University	Psychology	Mixed	Undergraduate	Intervention group (n = 141) Control group (n = 113)	1	01:00	Unknown
Lerchenfeldt *et al.*, 2020 [46]	USA	University	Nursing	2^nd^ year	Undergraduate	342	Unknown	Unknown	Unknown
Lu *et* *al.*, 2016 [65]	Taiwan	University	Nursing	Unknown	Undergraduate	Unknown	Unknown	Unknown	Unknown
Luebbert and Popkess, 2015 [47]	USA	University	Nursing	Mixed	Undergraduate	34 (simulation, n = 18) control group (lecture, n = 16)	1	01:40	Unknown
Magerman *et* *al*., 2022 [66]	Belgium	University	Interdisciplinary	4^th^ year	Undergraduate	14	3	01:00–01:40	Unknown
McKeirnan et al., 2023 [67]	USA	University	Pharmacy	Mixed	Postgraduate	235	1	07:30	Unknown
Mospan *et* *al*., 2017 [33]	USA	University	Interdisciplinary	1^st^ year	Undergraduate	356	15	03:00	Core
Muehlenkamp and Quinn-Lee, 2023 [42]	USA	University	Interdisciplinary	Mixed	Undergraduate	1345	1	00:40–00:50	Unknown
Muehlenkamp and Thoen, 2019 [26]	USA	University	Psychology and Social Work	Mixed – introduced at various stages of degree course	Undergraduate	68 (intervention group n = 38) (control group n = 30)	16	Unknown	Unknown
Nebhinani *et al*., 2020 [56]	India	University	Medicine	Mixed – introduced at various stages of degree course	Undergraduate	243	1	03:00	Unknown
Ng *et* *al*., 2022 [34]	New Zealand	University	Medicine	Mixed – introduced at various stages of degree course	Undergraduate	9	1	Unknown	Core
O’Reilly *et al.*, 2019 [48]	Australia	University	Pharmacy	3^rd^ year	Undergraduate	22	1	Unknown	Unknown
Osteen *et* *al*., 2014 [68]	USA	University	Social Work	2^nd^ year	Undergraduate	73	1	01:30	Unknown
Osteen, 2018 [45]	USA	University	Social Work	Unknown	Postgraduate	38	1	01:30	Unknown
Patel *et* *al*., 2018 [57]	India	University	Medicine	Unknown	Undergraduate	20	1	03:00	Unknown
Perez et *al*., 2022 [51]	USA	University	Nursing	Unknown	Postgraduate	105	1	00:15	Unknown
Phillips *et* *al*., 2019 [11]	United Kingdom	University – clinical placement	Medical Students	Mixed – introduced at various stages of degree course	Undergraduate	35	6	Unknown	Unknown
Pothireddy *et al*., 2022 [49]	USA	University	Pharmacy	Mixed – introduced at various stages of degree course	Undergraduate	139	1-3^i^	00:45	Unknown
Price *et* *al*., 2022 [35]	USA	University – clinical placement	Medicine	3^rd^ year	Undergraduate	75	1	Unknown	Core
Pullen *et al*., 2016 [36]	USA	University	Nursing	2^nd^ year	Undergraduate	150	1	01:30	Core
Quemada-González et al., 2024 [69]	Spain	University	Nursing	3^rd^ year	Undergraduate	72	1	Unknown	Unknown
Ranahan, 2020 [58]	Canada	University	Youth Work	Unknown	Undergraduate and Postgraduate	13	3	03:00	Unknown
Retamero *et* *al*., 2014 [50]	USA	University	Medicine	2^nd^ year	Undergraduate	180	1	01:36	Unknown
Scott, 2015 [70]	USA	University	Social Work	2^nd^ year	Postgraduate	20	15	01:30	Unknown
Sharpe *et* *al*., 2014 [43]	USA	University	Social work	2^nd^ year	Postgraduate	73 (intervention = 35) control (n = 33)	1	01:30	Unknown
Stallman, 2020 [59]	Australia	University	Interdisciplinary	Mixed	Undergraduate and Postgraduate	Unknown	8	Unknown	Unknown
Takahashi *et* *al*., 2022 [60]	Japan	University	Medicine	2^nd^ year	Undergraduate	136	3	01:15	Unknown
Vincent and Davis, 2016 [55]	Canada	University	Pharmacy	3^rd^ year	Undergraduate	1150	1	01:30	Unknown
Ward, 2011 [71]	Australia	University	Clinical Nursing	3rd year	Undergraduate	20	3	01:00	Unknown
Wathelet *et al*., 2023 [38]	France	University	Interdisciplinary	Mixed	Undergraduate	144 (intervention n = 48, control n = 96)	20	Mixed	Elective
Willson *et* *al*., 2020 [72]	USA	University	Pharmacy	1^st^ year	Undergraduate	136	Unknown	03:30	Unknown
Witry *et al*., 2020 [73]	USA	University	Pharmacy	Mixed – introduced at various stages of degree course	Undergraduate	111	2	02:30	Unknown
Witry *et* *al*., 2019 [74]	USA	University	Pharmacy	1^st^ year	Undergraduate	108	1	00:50	Unknown
Yousuf *et al*., 2013 [9]	China	University	Medicine and Surgery	Mixed – Elective	Undergraduate	22	10	05:30	Elective

^i^Taught in one session at one participating university and taught in three sessions at the second participating university.

Across the 58 included studies, there was limited reporting on whether suicide prevention education was delivered as a core (compulsory) or elective (optional) component of academic programmes. Only 13 studies (22%) explicitly indicated that the training was embedded as a required or core part of the curriculum [14,17,22,27–36]. In contrast, a small number of studies (*n* = 3) described the training as elective or optional, typically delivered through workshops, pilot modules, or extracurricular formats [9,37,38]. The majority of studies (*n* = 42, 73%) did not specify the course type. In several of these, training was delivered as a stand-alone intervention, a pilot programme, or through online modules, making it difficult to determine whether participation was voluntary or integrated into formal coursework.

Most articles did not specify the percentage of the module dedicated to suicide prevention, however, in some cases (*n* = 12, 20%), the full programme was dedicated to suicide prevention training [9,14,25–27,31,32,39–43]. Similarly, most papers did not mention a regulatory/accreditation body for the programme. Those that were mentioned included the Association of Directors of Medical Student Education in Psychiatry [44], the Quality Care Pharmacy Programme [28] and the American Psychiatric Association [9]. One of the programmes is listed in the National Registry of Evidence Based Programs and Practices [45]. Almost one quarter of articles (*n* = 13, 22%) outlined that the suicide prevention training programme was deemed essential and required training for their healthcare students [14,22,30,33–36,46–51] and the remainder either did not deem the coursework as essential or failed to specify. Eight articles (14%) described programmes that mandated attendance at the training [22,33–36,48–50] and the remainder either did not specify or did not require mandatory attendance.

### Learning outcomes

Typically, learning outcomes were linked to improving attitudes and fostering the acquisition of knowledge and skills, collectively referred to as core competencies. Overall, learning outcomes incorporated the acquisition of a range of abilities necessary for effectively identifying, assessing, and intervening in situations involving suicide risk. Learning outcomes for students typically involved learning about suicide statistics, risk factors, protective factors and warning signs and developing skills in risk screening and assessment and the implementation of an intervention for individuals with suicidal thoughts and behaviours. In one study, academic writing skills were included as learning outcomes for healthcare students in their suicide prevention training course [40]. In addition, two programmes included self-care elements as learning outcomes [40,52]. An overview of the aims of each of the programmes with the associated learning outcomes, where they were specified is provided in the supplementary information (S5 Table: Learning outcomes of suicide prevention training for health and social care students).

### Teaching methodologies

A wide range of methodologies were employed in the suicide prevention training programmes, as summarised in Table 2. Many studies reported varying methods of delivery and student preferences in relation to methodology, however, there is an apparent lack of evidence as to which are most effective in delivering suicide prevention content [44]. More than half of the programmes (*n* = 32, 55%) adopted a lecture-based format [11,14,17,23,25–27,29–31,33–35,38,41,44–47,49,52–60]. Role-playing exercises (n = 17, 29%) and patient simulations (n = 15, 25%) were also frequently incorporated into both delivery and assessment, while case studies (n = 6, 10%) were less commonly used. Discussion was a prominent method, with large group discussions featured in twelve programmes (20%) and small group discussions in ten programmes (17%). In one programme, medical students found skills training, including risk assessment and crisis intervention, and group discussions to be more beneficial over theory-based content [9]. Online learning was reported in nine studies (15%), and videos were frequently employed as a teaching tool (n = 17, 29%). Other methodologies included active learning (n = 4, 7%), interactive learning (n = 10, 17%) problem-based learning (n = 2, 3%), debate (n = 4, 7%), development of a research proposal (n = 2, 3%), readings, (n-9, 15%), self-reflection (n = 9, 15%) and think-pair-share (n = 1, 1.7%).

**Table 2:** Teaching methodologies.

Authors	Active Learning	Interactive Learning	Problem-based Learning	Case Studies	Debate	Development of a Research Proposal	Large Group	Small Group	Think-Pair-Share	Lecture	Online Learning	Patient Simulation	Pre-classroom Learning	Readings	Role Play	Self-Reflection	General Written Assignment	Video Clips	None Described
Almeida et al., 2017 [14]				✓	✓		✓	✓		✓	✓				✓				
Afsharnejad et al., 2022 [61]		✓		✓							✓							✓	
Bornheimer et al., 2024 [39]										✓		✓						✓	
Boukouvalas et al., 2018 [62]										✓		✓							
Carpenter et al, 2023 [37]							✓											✓	
Cates and Woolley, 2017 [5]																			✓
Chuop et al., 2020 [44]							✓			✓			✓						
Cramer et al., 2019 [40]		✓						✓						✓		✓			
Cramer & Long 2018 [27]		✓			✓					✓	✓			✓		✓			
Cramer et al., 2016 [52]					✓	✓				✓					✓				
Desai et al., 2018 [53]										✓					✓				
De Silva et al., 2015 [17]				✓			✓			✓					✓			✓	
El-Den et al., 2018 [28]												✓			✓				
Goh et al., 2016 [29]										✓		✓							
Harshe et al., 2022 [30]										✓					✓				
Heyman et al., 2015 [31]							✓			✓					✓				
Hjelvik et al., 2022 [25]							✓			✓					✓				
Hill et al., 2024 [63]											✓								
Hutson and Zeno, 2021 [54]	✓			✓						✓		✓				✓			
Jacobson et al., 2012 [41]										✓									
Kerr et al., 2018 [32]							✓						✓					✓	
Kourgiantakis et al., 2021 [24]	✓	✓										✓					✓	✓	
Kullberg et al., 2020 [64]				✓							✓							✓	
Kratz et al., 2020 [23]										✓		✓		✓	✓				
LeCloux, 2021 [22]																			✓
Lerchenfeldt et al., 2020 [46]				✓			✓			✓			✓	✓	✓	✓			
Lu et al., 2016 [65]												✓				✓		✓	
Luebbert and Popkess, 2015 [47]										✓		✓							
Magerman et al., 2022 [66]								✓			✓				✓	✓		✓	
McKeirnan et al., 2023 [67]								✓			✓		✓						
Muehlenkamp and Quinn-Lee, 2023 [42]																			✓
Mospan et al., 2017 [33]		✓						✓		✓		✓							
Muehlenkanp and Thoen, 2019 [26]	✓	✓					✓			✓									
Nebhinani et al., 2020 [56]										✓									
Ng et al., 2022 [34]								✓		✓	✓							✓	
O’Reilly et al., 2019 [48]												✓							
Osteen, 2018 [45]										✓									
Osteen et al., 2014 [68]																			✓
Patel et al., 2018 [57]		✓								✓									
Perez et al., 2022 [51]												✓							
Phillips et al., 2019 [11]							✓			✓		✓				✓			
Pothireddy et al., 2022 [49]										✓				✓		✓		✓	
Price et al., 2022 [35]										✓		✓							
Pullen et al., 2016 [36]														✓					✓
Quemada-González et al., 2024 [69]			✓									✓							
Ranahan, 2020 [58]										✓					✓	✓		✓	
Retamero et al., 2014 [50]																			
Scott, 2015 [70]						✓		✓						✓			✓	✓	
Sharpe et al., 2014 [43]																			✓
Stallman, 2020 [59]		✓								✓				✓					
Takahashi et al., 2022 [60]										✓					✓			✓	
Vincent and Davis, 2016 [55]				✓	✓					✓					✓				
Ward, 2011 [71]			✓	✓				✓		✓								✓	
Wathelet et al., 2023 [38]										✓					✓				
Willson et al., 2020 [72]	✓			✓			✓				✓		✓					✓	
Witry et al., 2020 [73]				✓			✓								✓				
Witry et al., 2019 [74]		✓		✓				✓	✓				✓	✓	✓				
Yousuf et al., 2013 [9]		✓		✓				✓										✓	

### Evaluations of suicide prevention training programmes

All studies in this review that included student evaluations (n = 32, 55%) reported that most health and social care students found suicide prevention content to be positive, valuable, and beneficial. Table 3 outlines the findings from studies that included an evaluation of an implemented programme. Negative outcomes for students were not specifically reported considering the training, although the content was considered emotionally difficult by some participants [73].

**Table 3:** Evaluation of suicide prevention training programmes.

Author (year)	Study design	Follow-up periods	Measures	Type of control group	Outcomes
Almeida et al., 2017 [14]	Pre-post evaluation	Immediately before and after the module	Knowledge, confidence, and preparedness	N/A	Statistically significant increases in knowledge, confidence, and preparedness from pre-test to post-test. Knowledge, confidence, and preparedness were significantly positively correlated, indicating that confidence and preparedness did not increase without a corresponding increase in knowledge.
Afsharnejad et al., 2022 [61]	Randomised control trial	Data was collected at three time points: baseline, 10-weeks, and 24-weeks from baseline.	Suicide Intervention Response Inventory (SIRI)	Delayed start group	Intention-to-treat analysis (N = 129) at 10-weeks demonstrated a significant improvement in generalised self-efficacy for “Talk-to-Me” compared to the control group with only the “Talk-to-Me” participants reporting increased knowledge in responding to suicidal ideation (primary outcome). This change was sustained for 24 weeks.
Bornheimer et al., 2024 [39]	Pre-post evaluation	Immediately before and after the module	Acceptability, feasibility, and preparedness	N/A	Simulations were reported to be acceptable and feasible, with strong student desire and need for greater suicide prevention training. Significant improvements were reported in clinical skills via simulated training scores and perceptions of clinical preparedness.
Carpenter et al., 2023 [37]	Pre-post evaluation	Immediately before and after the module	Knowledge and self-efficacy	N/A	Both the mean knowledge score and mean self-efficacy score significantly increased from pre-test to post-test.
Cates et al., 2017 [5]	Pre-post evaluation	Immediately before and after the clinical placement	Attitudes to Suicide Prevention Scale	N/A	Statistically significant positive changes in total scale scores from pre-rotation to post-rotation were seen in attitudes toward suicide prevention.
Choup et al., 2021 [44]	Pre-post evaluation	Immediately before and after the module	Self-report, pre-post survey was developed utilizing Ajzen’s Theory of Planned Behaviour measurement framework	N/A	Participants showed significant improvements in attitudes, confidence, and intentions towards using suicide prevention strategies, with medium-to-large effect sizes overall. The greatest improvements were in familiarity with suicide risk assessment, access to lethal means, and safety planning. The smallest improvements were in competency for identifying suicidal patients and understanding the role of medical providers in suicide prevention.
Cramer et al., 2019 [40]	Pre-post evaluation	Immediately before and after the module	Suicide competency assessment form Suicide Behaviours and Attitudes Questionnaire Scale Suicide Prevention Knowledge Quiz IPECC-Set	N/A	Primary outcomes included: (1) significant moderate-to-large gains in suicide prevention knowledge, clinical care skills, and ability to help self-harming patients; (2) moderate improvements in sensitivity to suicide risk factors; (3) non-significant impacts on IPE-related outcomes.
Cramer and Long, 2018 [27]	Pre-post evaluation	Immediately before and after the module	1 A ten-question multiple-choice knowledge quiz 2 Suicide competency assessment form	N/A	Significant positive gains in suicide prevention knowledge, and self-perceived suicide prevention competency total score.
Cramer et al., 2016 [52]	Pre-post evaluation	Immediately before and after the module	Suicide Intervention Response Inventory-2 (SIRI-2) Attitudes Toward Suicide Prevention Scale Suicide competency assessment form Suicide risk assessment knowledge quiz	N/A	Significant increase in knowledge of suicide risk assessment and management, as well as improved student accuracy in estimating chronic and acute suicide risk in response to a mock case vignette.
De Silva et al., 2015 [17]	Pre-post evaluation	Immediately before and after the module	Knowledge, skills, and attitudes	N/A	Findings reported an increase in knowledge, skills and attitudes related to the assessment and management of individuals at risk for suicide, and the application of this ability to students’ personal and professional lives.
Desai et al, 2018 [53]	Pre-post evaluation	Immediately before and after the intervention	Knowledge, skills, and attitudes	N/A	The intervention was found effective in increasing knowledge, changing attitude, and enhancing communication skills of medical students toward suicide prevention.
Goh et al., 2016 [29]	Pre-post evaluation	Immediately before and after the intervention	Confidence and satisfaction	N/A	The use of standardised patient sessions significantly increased students’ satisfaction and confidence. Qualitative feedback from students indicated a positive view of standardised patients as an effective tool for enhancing practical skills alongside didactic learning.
Harshe et al., 2022 [30]	Pre-post evaluation	Immediately before and after the workshop	Suicide Opinion Questionnaire (SOQ)	N/A	There was a 9.5% increase in SOQ scores post-intervention indicating a change toward positive attitudes/beliefs about suicide.
Hjelvik et al., 2022 [25]	Pre-post evaluation	Immediately before and after the workshop	A self-report pre-/postsurvey using Ajzen’s theory of planned behaviour framework	N/A	Pre-/post surveys showed the greatest improvements in suicide prevention knowledge (self-rated) and the confidence in and likelihood of asking peers about suicide.
Hill et al., 2024 [63]	Randomised control trial	Immediately before and after the training	Literacy of Suicide Scale (LOSS) Gatekeeper Behaviour Scale Attitudes Toward Suicide Prevention Scale (ATSPS)	Control group received a suicide prevention module that purposefully omitted key elements of gatekeeper training (i.e., the AS+K? skills)	Compared to those in the control group, participants in the intervention group reported significantly greater gatekeeper preparedness and self-efficacy, and lower stigmatising attitudes towards suicide post on completion of the training. There were no significant differences in the likelihood of utilizing gatekeeper skills or suicide-related knowledge between the groups.
Jacobson et al., 2012 [41]	Randomised control trial	Immediately after training and 6 months later	1 Knowledge towards suicide prevention 2 Attitudes to Suicide prevention (ASP) Scale 3 Perceived preparedness 4 Asking Clients about Suicide in Response to Warning Signs	Delayed start group	Overall, most students who completed the QPR training were satisfied with it and reported they would recommend the training to a peer or colleague. Interaction effects between group assignment and time suggest improvement among the intervention group regarding knowledge, efficacy to perform the gatekeeper role, and skills. Both groups improved over time for reluctance to engage with clients at risk for suicide, referral, and gatekeeper behaviours.
Kerr et al., 2018 [32]	Pre-post evaluation	Immediately before and after the training	General Perceived Self-efficacy (GPSE) Scale	N/A	The results of the study show that the SafeTALK training had a positive impact on increasing the general self-efficacy of the participants in the whole sample. Both Males and females reported increased self-efficacy post SafeTALK training. This reported increase was more marked in the males in the sample compared to the females.
Kratz et al., 2022 [23]	Pre-post evaluation	Immediately before and after the training	1 Knowledge survey 2 Counselling Self-Estimate Inventory	N/A	Results indicate statistically significant improvements in students’ knowledge and CSEI scores overtime. Moreover, CSEI and the subscale of dealing with difficult client behaviours showed statistically significant improvements from pre-simulation to post-simulation.
Kullberg et al, 2020 [64]	Randomised control trial	1- and 3-months post intervention	Knowledge, confidence, and guideline adherence	Delayed start control	Intention-to-treat analysis showed that the students in the intervention condition reported higher levels of self-evaluated knowledge, provider’s confidence, and guideline adherence than those in the waitlist control.
LeCloux, 2021 [22]	Pre-post evaluation	Immediately before and after the training	Knowledge, preparedness, and confidence	N/A	Scores for suicide-related knowledge, perceived preparedness, and confidence increased significantly from pre- to post-test. Most students (95.7%) were satisfied or highly satisfied with the overall content, comprehensiveness, and format of the module. However, most (70.2%) reported a preference for face-to-face or hybrid delivery methods for suicide-related material rather than a fully online model.
Muehlenkamp and Quinn-Lee, 2023 [42]	Pre-post evaluation	Pre- post- and 3-month follow-up	Knowledge, skills, intention to intervene and self-efficacy through the “Program impact survey”	N/A	Participants showed substantial increases in all outcomes from pre- to post-training, and these gains were maintained at follow-up
Muehlenkamp and Thoen, 2019 [26]	Quasi- experimental	Pre- post- and 4-month follow-up	1 Stigma of Suicide (SOSS) Scale 2 Suicide Prevention Advocacy	Students in the courses Cognitive Basis of Religion or Illness and Identity	Students in the suicidology course showed significant pre- to post- increases in knowledge and suicide prevention advocacy, alongside reductions in suicide stigma and negative attitudes compared to students in the control course, who showed no significant pre-/post changes. All effects were maintained over time.
Nebhinani et al., 2020 [56]	Pre-post evaluation	Immediately before and after the workshop	Attitudes to suicide prevention	N/A	Ten out of 14 attitudinal statements were significantly more favourable after completing a brief training on suicide prevention and management of suicide attempters.
Osteen, 2018 [45]	Randomised control trial	Pre- post- and 4-month follow-up	Knowledge, attitudes, self-efficacy, and gatekeeper behaviours.	Matched control group	Results suggest improvements in post training measures for knowledge, attitudes, self-efficacy, reluctance, and the use of gatekeeper behaviours, but there was no supporting evidence for the presence of mediated effects on behaviour. Only self-efficacy demonstrated a strong direct relationship with gatekeeper behaviours.
Patel et al., 2018 [57]	Pre-post evaluation	Immediately before and after the workshop	Knowledge, attitudes, and skills	N/A	This study compared the knowledge, attitudes, and skills of both students and teachers after completing a suicide prevention gatekeeper training. Undergraduate students developed more positive attitude for suicidal behaviour where faculties developed more confident in their skill after training sessions.
Pothireddy et al., 2022 [49]	Pre-post evaluation	Immediately before and after the training	Knowledge and confidence	N/A	Students’ confidence and knowledge in recognizing and managing suicide warning signs improved significantly. More students directly asked about suicide and expedited referrals. Most (86%) reported planning to incorporate what they learned into practice
Pullen et al., 2016 [36]	Pre-post evaluation	Immediately before and after the training	Knowledge of suicide prevention	N/A	Overall, students responded very positively to suicide prevention gatekeeper training. Participants showed significant improvements in knowledge of suicide prevention pre‐ to post‐training.
Stallman, 2020 [59]	Pre-post evaluation	Immediately before and after the training	Knowledge, attitudes, confidence, and self-care	N/A	Participants showed significant improvements in knowledge, attitudes, confidence, and self‐care pre‐ to post‐training with moderate to very large effect sizes. There was no significant difference in outcomes between those who had and had not had previous training or experience working with people with suicidality.
Wathelet et al., 2023 [38]	Quasi-experimental	Immediately before and after the intervention	Suicide Attitudes and Behaviours (SABQ) Questionnaire	Matched control group assigned to other modules	Compared to the unexposed group, the exposed group reported greater satisfaction with the training, improved self-confidence in professional capacities, and a higher likelihood of having identified or helped someone with a mental health issue or consulted about a mental health concern.
Wilson et al., 2020 [72]	Pre-post evaluation	Immediately before and after the training	Attitudes, knowledge, and confidence	N/A	Students’ comfort level with asking about suicidal ideation and their confidence with intervening significantly increased from the pre- to post-intervention survey.
Witry et al., 2020 [73]	Pre-post evaluation	Immediately before and after the module	Knowledge, confidence, and intention to intervene	N/A	Students showed significant improvements in confidence and knowledge related to suicide prevention. Three-quarters (73%) reported being very or extremely likely to intervene when seeing warning signs, with confidence strongly linked to this likelihood. Most (93%) felt the training provided the right amount of background information, though 43% wanted more practice. Additionally, 35% found the material moderately emotionally difficult, and 5 students found it very or extremely emotionally difficult.
Yousuf et al, 2013 [9]	Pre-post evaluation	Immediately before and after the module	CASQ-HK Scale (attitudes and confidence in identifying a suicidal patient)	N/A	Participation in the module led to statistically significant changes in attitudes towards suicide, including reduced negative appraisal, decreased stigmatization, and increased sensitivity to suicide-related facts.

Of these studies, the most commonly assessed outcomes were levels of knowledge (n = 22/32, 69%) [14,17,22,23,25–27,36,37,40–42,45,49,52,53,57,59,61,63,64,72], confidence (n = 11/32, 34%) [14,22,25,29,38,44,49,59,64,72,73], self-perceived self-efficacy or competency (n = 9/32, 28%) [23,26,27,37,41,45,61,63] and attitudes towards suicide/suicide prevention (n = 8/32, 25%) [5,9,17,26,44,45,57,59]. Many programmes demonstrated significant improvements in students’ knowledge and confidence regarding suicide prevention. For instance, Almeida et al. (2017) reported statistically significant increases in knowledge, confidence, and preparedness following their module [14]. Similarly, Carpenter et al. (2023) and Witry et al. (2020) found substantial gains in both knowledge and self-efficacy post-training [37,73], with a notable number of students feeling more prepared to intervene when recognising warning signs.

Several evaluations emphasised the importance of practical training methods, such as simulations and role-playing. Bornheimer et al. (2024) and Cramer et al. (2019) found that simulation-based training significantly improved clinical skills and preparedness [39,40]. Students consistently expressed a desire for more hands-on training, and programs utilising standardised patients and case-based scenarios often reported higher satisfaction rates [29]. Many students indicated a preference for interactive and practical learning methods over theoretical instruction. LeCloux (2021) found that most students preferred face-to-face or hybrid delivery models [22], and several studies highlighted improvements in competency through active participation in suicide prevention strategies [22,29,39,40,44,73].

### Challenges associated with suicide prevention training programmes

Stigma and the difficulty discussing the topic of suicide was reported as a challenge for students and faculty in one study [72]. This was also evidenced in skills demonstration as many students did not directly ask about suicide during role play activities despite the training provided. One study of a suicide prevention training programme with pharmacy students noted that it was challenging to encourage students to apply their suicide prevention training skills following a training session [73]. However, the authors presented the idea of employing the use of patient simulations as a possibility for providing students with the opportunity to practise applying the skills. The need for students to apply their knowledge and skills was also highlighted by Scott et al. (2015) who reported that students are eager to engage in deeper learning and to learn ‘what to say when…’ as well as the general risk and protective factors associated with suicide prevention [70]. Additionally, Cramer and Long (2018) reported that the inclusion of people with lived experience for patient simulation provides an avenue for further interaction for students to apply their skills in a meaningful way [27]. However, further research on this is warranted [23].

A recurring challenge identified was the limited time available to cover all necessary content [11,17,36,52], in particular, the importance of allowing sufficient time for students to practise applying their new skills in suicide prevention [44]. Another significant barrier citied was the lack of space within the existing curriculum, which often relegates suicide prevention training to elective courses rather than required coursework To address this, Cramer and colleagues (2016) suggested integrating suicide prevention content into existing modules, such as psychological assessment, abnormal psychology, or counselling theories, ensuring that it becomes a core component of healthcare education [52].

## Discussion

This scoping review identified 58 articles describing the design, development, implementation, and/or quantitative evaluation of suicide prevention training for health and social care students, presenting findings from across thirteen countries. In summary, suicide prevention training was associated with increasing students’ knowledge, confidence, and favourable attitudes towards suicide prevention; however, the content of the programmes, as well as the development and modality of the training, varied significantly. In stating this, it is important to acknowledge the need for flexibility in suicide prevention training. This adaptability is a key consideration, as it demonstrates that institutions can tailor suicide prevention training to fit their specific needs and constraints. This aligns with broader literature on curricular innovation, which emphasises the importance of agile, flexible, context-sensitive approaches in healthcare education [75,76]. Whether offering a single session or a semester-long course, institutions can design programmes that fit within their curricular framework, ensuring that suicide prevention content can be delivered, even in resource-limited environments.

In articles that had specified learning outcomes, the focus was centred on developing core competencies related to suicide prevention – recognising risk and protective factors, communication, and intervention skills. These outcomes were consistent across healthcare disciplines, highlighting the essential skills required to identify, assess, and intervene with individuals at risk regardless of profession. These competencies align with frameworks such as the UK’s National Collaborating Centre for Mental Health suicide prevention competency framework, which aim to support professionals without formal mental health training [77]. It seeks to bridge gaps in knowledge and provide practical information, enabling healthcare professionals to contribute effectively to suicide prevention efforts. Comparable frameworks have been recommended in international guidance, including WHO (2014), suggesting that suicide prevention training should be embedded as a universal competence for all frontline professionals [78]. Indeed, these core competencies are not unique to suicide prevention. Similar skills are required in other priority areas of healthcare education, including domestic violence response, substance use intervention, and trauma-informed care [79–81]. All of these domains require professionals to recognise complex risk factors, communicate compassionately in high-stakes situations, and take appropriate action – often without the benefit of specialist training. Embedding these transferable skills across curricula ensures that future practitioners are well-equipped to respond confidently and ethically to a range of psychosocial challenges encountered in clinical practice.

The use of diverse teaching methodologies, such as case studies, group discussions, and role-play, reflects efforts to make suicide prevention training more engaging and practical, highlighting issues such as health inequalities and cultural sensitivities. There is, however, no consensus on the most effective delivery methods to use [44]. While lectures are a common component, which are important in imparting foundational knowledge, they appear to be less effective compared to interactive strategies such as role-playing and patient simulations, which provide students with hands-on experience in crisis intervention [82]. A study reflecting on five years of suicide prevention training for pharmacy students found that the most effective methods to train students in crisis intervention involved both inspiring them and equipping them with practical interview techniques to confidently ask about suicide [53]. This aligns with educational research in other areas of health and social care, where several studies have found that active learning methods, including patient simulations, are regarded as valuable learning tools that students can effectively apply in clinical practice [83–91]. The inclusion of teaching methodologies that facilitate experiential learning are supported by Kolb’s Experiential Learning Theory, which emphasises that learning through transformation of experience consolidates active learning and reflection [92]. These approaches facilitate deeper engagement and long-term knowledge retention, especially when addressing sensitive topics such as suicide [93,94].

The inclusion of people with lived experience in patient simulations was also noted in the literature for its positive impact on students’ confidence and communication skills. For example, one study reporting that involving people with lived experience as simulated patients resulted in sustained improvements in student confidence in discussing suicidal behaviour with those at risk [62]. These findings mirror outcomes seen in other healthcare education domains, where co-production with service users enhances empathy, communication, and learner engagement [95,96]. However, the literature also highlights the need for further research into the ethical, emotional, and pedagogical dimensions of this practice, to ensure that the inclusion of lived experience is both meaningful and safe for all involved [97].

The international scope of the literature included in this review reflects the global recognition of suicide prevention as a crucial competency for all health and social care professionals. Moreover, the inclusion of interdisciplinary teaching in 17% (*n* = 10) of the programmes signifies a growing awareness that suicide prevention is relevant across healthcare and social care disciplines. By identifying programmes that incorporate several healthcare disciplines, this review demonstrates the recognised importance of collaborative, cross-disciplinary teaching approaches. Research shows that interprofessional education enhances collaboration, mutual respect, and holistic understanding of complex issues like suicide prevention [98,99] Consequentially, the integration of interprofessional education into training programmes can enhance the competencies of future practitioners, enabling them to better understand the multifaceted nature of suicide risk factors and the diverse needs of at-risk populations [40]. Interprofessional learning experiences encourage students to engage in shared problem-solving and develop mutual respect for each profession’s contributions, leading to more effective care strategies [100,101]. However, interprofessional education remains an underutilised strategy in suicide prevention education and curricula [4,40]. As the landscape of healthcare continues to evolve, ongoing research into best practices for interprofessional education in suicide prevention will be critical. Future curriculum development should build on interprofessional frameworks to prepare students for real-world, team-based clinical contexts.

Several challenges in the development and implementation of suicide prevention training were highlighted across the literature. One significant barrier was the stigma associated with discussing suicide in educational settings [72]. However, educational engagement with the topic was found to contribute to a reduction in stigmatising attitudes [56,102,103], echoing broader mental health education literature that supports the role of structured learning in shifting beliefs and promoting attitudinal change [104,105]. Furthermore, the inclusion of suicide prevention training was also observed to be associated with an increase in help-seeking behaviour from the students themselves. This is an encouraging finding, as there is a widely recognised risk of suicide in certain occupational groups including healthcare professionals [106,107]. This underscores the need for a structured self-care component in curricula, led by those with expertise in the area which presents a practical challenge. Learning outcomes for the topic of self-care were observed in only two of the included studies, showing a significant gap in addressing this need. Embedding this within suicide prevention training would align with wider calls in health education for curricula that support both clinical competency and practitioner wellbeing [108].

A key issue identified in the evaluation data of the included literature was a lack of long-term follow-up. While improvements in knowledge, confidence, and self-efficacy are highlighted, there is little discussion of whether these gains are sustained over time or translated into practical application in clinical settings. This gap points to the need for future research assessing the long-term impact of suicide prevention training on clinical practice and patient outcomes.

Another more practical challenge observed in the literature was identifying sufficient time in the student curriculum to facilitate training, an issue [11,17,52]., which also arises in relation to other course offerings for health and social care disciplines [109]. This needs to be addressed at local level to ensure feasibility and appropriateness of suicide preventing training to respective curricula.

Although the review includes studies from multiple countries, it is heavily dominated by those from the USA and Australia. This may limit the relevance of the findings for countries with different healthcare systems, educational structures, or cultural attitudes towards suicide. As a result, the conclusions may not fully reflect the needs or experiences of institutions in lower-income regions or those with different public health priorities. This under-representation may reflect a combination of structural factors, including limited academic output, lack of formal suicide prevention education in LMICs, and resource constraints that hinder curriculum development or evaluation. For instance, Doty et al. (2022) conducted a systematic review and found that most suicide prevention studies in LMICs were concentrated in a few countries, with a notable scarcity of large-scale investigations tailored specifically for youth [110]. Additionally, the World Health Organization (WHO) has emphasised that many LMICs lack national suicide surveillance systems and systematic reporting, which hampers the development and evaluation of suicide prevention strategies [111]. As a result, the findings of this review may disproportionately reflect practices in high-income contexts. Future efforts should prioritise investment in suicide prevention training in LMICs, support capacity-building for local research, and encourage the development of culturally and contextually appropriate curricula. International collaboration and funding mechanisms could also play a role in bridging this evidence gap and ensuring more inclusive and globally relevant suicide prevention education.

### Strengths and limitations

The review’s broad inclusion criteria, covering multiple healthcare disciplines, geographical regions, and both undergraduate and postgraduate programmes, ensures a comprehensive overview of suicide prevention training. This wide scope allows for a more inclusive summary of current practices, making the findings applicable to a variety of healthcare disciplines. Moreover, by encompassing both small-scale and large-scale programmes, the review captures a diverse range of teaching approaches, student populations, and institutional practices, providing a more complete picture of the current suicide prevention training landscape.

Another significant strength of this scoping review was the methodological rigor applied to the search strategy, which we continuously sought to update. Additionally, we made a concerted effort to explore as many grey literature sources as possible within our time constraints, and we also conducted both forward and backward citation searching. A further strength lies in the decision to include only peer-reviewed publications, ensuring that all sources met recognised academic standards and had undergone formal scholarly review. This approach enhances the credibility and methodological rigour of the findings presented. However, this decision also presents a limitation. By excluding non-peer-reviewed grey literature – such as policy documents, national frameworks, and institutional curriculum drafts – we may have missed valuable insights into the practical development and implementation of suicide prevention curricula, particularly in low- and middle-income countries where academic publication may be less frequent. Future reviews may benefit from incorporating high-quality grey literature to capture a more comprehensive picture of global educational practices in this area.

A further notable limitation is the variability in the level of detail reported across the included studies. Many articles did not specify key elements, such as the proportion of the programme devoted to suicide prevention, the involvement of regulatory or accreditation bodies, or detailed descriptions of the teaching methodologies used. This inconsistency in reporting makes it difficult to draw clear conclusions about the structure and content of these programmes and hampers the ability to compare studies effectively.

Additionally, the review does not provide a thorough assessment of the quality of the studies included. As scoping reviews aim to map the existing literature rather than critically appraise study quality, this approach can lead to the inclusion of studies with varying levels of methodological rigour. Equally important to consider, is the focus of this review on module design and quantitative outcomes, excluding insights gained from qualitative findings. We acknowledge that there is much to learn from student experiences and perceptions of suicide prevention training [9,31,43]. Future work should look to focus on culmination of qualitative research to better understand students’ experiences, preferences and feedback on such training.

## Conclusion

In synthesis, by embedding a standardised set of competencies across all disciplines in an interprofessional setting, students can develop the necessary foundational skills for identifying and responding to those at risk. Despite the development of learning outcomes to reflect core competencies in suicide prevention within the reviewed literature, considerable variability has been noted. The absence of standardised training and competency frameworks across institutions and countries reflects ongoing challenges in ensuring that all professionals prepared to address suicide risk. A unified framework would not only ensure that all professionals are equally equipped in suicide prevention skills, but also promote interprofessional collaboration, enabling a more cohesive and coordinated approach to suicide prevention education. This review is an important first step in cohering the available evidence to inform the development of standardised competencies for suicide prevention training for health and social care students.

## Supporting information

S1 Table PRISMA ScR Checklist. (DOCX)

S2 Table Search strategy and results. (DOCX)

S3 File Indexes in Web of Science Core Collection. (DOCX)

S4 Table Data extraction template. (DOCX)

S5 Table Learning outcomes of suicide prevention training for health and social care students. (DOCX)
