# Supplementary material for: Suicide prevention curriculum development for health and social care students: A scoping review
Source: PLoS One. 2025 Jul 28;20(7):e0328776. doi: 10.1371/journal.pone.0328776 (PMC12303339; doi:10.1371/journal.pone.0328776)
Supplement: S2 Table — (DOCX) [file pone.0328776.s002.docx]

S2 Table: Search strategy and results

**Embase Search Strategy and Results**

| No. | Query | Results | Date |
| --- | --- | --- | --- |
| #23 | #1 AND #14 AND #21 AND [english]/lim AND [2011-2024]/py | 513 | 05-Jul-24 |
| #22 | #1 AND #14 AND #21 | 635 | 05-Jul-24 |
| #21 | #15 OR #16 OR #17 OR #18 OR #19 OR #20 | 586212 | 05-Jul-24 |
| #20 | postgraduate*:ti,ab,kw | 35834 | 05-Jul-24 |
| #19 | undergraduate*:ti,ab,kw | 69756 | 05-Jul-24 |
| #18 | 'student*':ti,ab,kw | 505912 | 05-Jul-24 |
| #17 | 'social care student*':ti,ab,kw | 64 | 05-Jul-24 |
| #16 | 'healthcare student*':ti,ab,kw | 1567 | 05-Jul-24 |
| #15 | 'health student'/exp | 153808 | 05-Jul-24 |
| #14 | #2 OR #3 OR #4 OR #5 OR #6 OR #7 OR #8 OR #9 OR #10 OR #11 OR #12 OR #13 | 5086335 | 05-Jul-24 |
| #13 | course:ti,ab,kw | 955851 | 05-Jul-24 |
| #12 | syllabus:ti,ab,kw | 1858 | 05-Jul-24 |
| #11 | program*:ti,ab,kw | 1536855 | 05-Jul-24 |
| #10 | curricul*:ti,ab,kw | 103222 | 05-Jul-24 |
| #9 | 'curriculum'/exp | 115307 | 05-Jul-24 |
| #8 | learning:ti,ab,kw | 692914 | 05-Jul-24 |
| #7 | teaching:ti,ab,kw | 254024 | 05-Jul-24 |
| #6 | training:ti,ab,kw | 803762 | 05-Jul-24 |
| #5 | 'medical education':ti,ab,kw | 78669 | 05-Jul-24 |
| #4 | 'medical education'/exp | 412931 | 05-Jul-24 |
| #3 | educat*:ti,ab,kw | 1117503 | 05-Jul-24 |
| #2 | 'education'/exp | 1801839 | 05-Jul-24 |
| #1 | 'suicide prevention'/exp OR 'suicide prevention' | 11393 | 05-Jul-24 |
