# Supplementary material for: Suicide prevention curriculum development for health and social care students: A scoping review
Source: PLoS One. 2025 Jul 28;20(7):e0328776. doi: 10.1371/journal.pone.0328776 (PMC12303339; doi:10.1371/journal.pone.0328776)
Supplement: S3 File — (DOCX) [file pone.0328776.s003.docx]

S3 File: Indexes in Web of Science Core Collection

**Web of Science Core Collection Indexes and Editions**

The Core Collection is the main curated set of publications in Web of Science. The Editions (indexes) are the specific databases within the Core Collection, each covering different disciplines and types of publications.

**Included in the authors institutional subscription:**

- BIOSIS Citation Index
- BIOSIS Previews
- Current Contents Connect
- Data Citation Index
- Derwent Innovations Index
- Grants Index
- KCI-Korean Journal Database
- MEDLINE®
- Preprint Citation Index
- ProQuest ™ Dissertations & Theses Citation Index
- SciELO Citation Index
- Zoological Record

**Included in the Editions**

- Science Citation Index Expanded (SCI-EXPANDED)--1945-present
- Social Sciences Citation Index (SSCI)--1956-present
- Arts & Humanities Citation Index (AHCI)--1975-present
- Conference Proceedings Citation Index – Science (CPCI-S)--1990-present
- Conference Proceedings Citation Index – Social Science & Humanities (CPCI-SSH)--1990-present
- Book Citation Index – Science (BKCI-S)--2005-present
- Book Citation Index – Social Sciences & Humanities (BKCI-SSH)--2005-present
- Emerging Sources Citation Index (ESCI)--2020-present
- Current Chemical Reactions (CCR-EXPANDED)--1985-present
- Index Chemicus (IC)--1993-present

On the search page the drop-down menu lists the databases and editions used: <https://www-webofscience-com.ucc.idm.oclc.org/wos/woscc/basic-search>

Our Web of Science subscription is part of the national IRIS Consortium of Irish University and Research Libraries.
