# Supplementary material for: Suicide prevention curriculum development for health and social care students: A scoping review
Source: PLoS One. 2025 Jul 28;20(7):e0328776. doi: 10.1371/journal.pone.0328776 (PMC12303339; doi:10.1371/journal.pone.0328776)
Supplement: S4 Table — (DOCX) [file pone.0328776.s004.docx]

S4 Table: Data extraction template

| Author, Year | DOI | Country | Setting | Degree course(s) | Stage of the Degree | Student Population | Name of the programme | Number of Students | Number of Sessions | Session Length | Does the content feature as part of a larger module? | % of module dedicated to Suicide Prevention | Accreditation Body | Is the training deemed 'essential' or required? | Attendance Mandatory | How many facilitators delivered the module/training? | Did the staff require additional training? | How was the programme implemented? | What are the learning outcomes? | Programme details/Content | What methodologies were used? | Assessment | Primary aim of the study | Main Finding | Intervention group details | Type of control | Length of Follow-up | Mean and standard deviation of all study groups in the relevant outcomes at all assessment times |
| --- | --- | --- | --- | --- | --- | --- | --- | --- | --- | --- | --- | --- | --- | --- | --- | --- | --- | --- | --- | --- | --- | --- | --- | --- | --- | --- | --- | --- |
|  |  |  |  |  |  |  |  |  |  |  |  |  |  |  |  |  |  |  |  |  |  |  |  |  |  |  |  |  |
|  |  |  |  |  |  |  |  |  |  |  |  |  |  |  |  |  |  |  |  |  |  |  |  |  |  |  |  |  |
