# Supplementary material for: Suicide prevention curriculum development for health and social care students: A scoping review
Source: PLoS One. 2025 Jul 28;20(7):e0328776. doi: 10.1371/journal.pone.0328776 (PMC12303339; doi:10.1371/journal.pone.0328776)
Supplement: S5 Table — (DOCX) [file pone.0328776.s005.docx]

S5 Table:

**Learning outcomes of suicide prevention training for health and social care students**

| Authors | Name of Programme | Aim of Programme | What are the learning outcomes? |
| --- | --- | --- | --- |
| Almeida et al. (2017) | Understanding Suicide: Prevention, Intervention, and Postvention | To develop and improve screening, assessment, and management of suicidal clients and of postvention and to provide information on suicide from a public health approach | - To understand the epidemiology and theories of suicide, as well as the various models of suicide prevention - To understand risk and protective factors (at multiple levels) for suicide and identify and understand which social groups are at high risk for suicide - To critically evaluate, select, and apply evidence-based suicide risk screening and assessment - To learn about development and implementation of interventions for individuals with suicidal thoughts and behaviours |
| Afsharnejad et al., 2022 | Talk-to-Me | To teach students to identify and respond to suicidal crises and suicidal thoughts and behaviours | Not specified |
| Bornheimer et al., 2024 | Not specified | Not specified | Not specified |
| Boukouvalas et al. (2018) | Mental Health First Aid (MHFA) | To improve attitudes towards and confidence in responding to patients with suicidal ideation | Not specified |
| Carpenter et al, 2023 | Pharm-SAVES | Not specified | Not specified |
| Cates and Woolley, 2017 | Advanced Pharmacy practice | Not specified | Not specified |
| Chuop et al. 2021 | N/A -- content delivered as part of the second-year Nervous System and Function module | To introduce clinical suicide prevention skills earlier in medical education and to provide a foundation for learning suicide-safer care (assess, intervene and monitor) | - To learn information on suicide statistics, risk factors, protective factors, and warning signs - To understand how to use standardized tools for risk screening - To learn basic components of risk assessment - To learn about evidence-based interventions, and tips on helpful communication skills - To apply safety interventions including collaborative safety planning, counselling on access to lethal means, appropriate triage, and referral for acute or specialty care, application of caring contacts, and appropriate interval follow-up |
| Cramer et al. (2019) | Suicide prevention and interprofessional education | To improve self-reflection and clinical care skills in suicide prevention | - To manage personal suicide-related attitudes - To establish an empathic rapport - To elicit risk/protective factors - To assess current suicidal intent and plan - To determine suicide risk level - To enact an evidence-based treatment plan - To involve social support - To adhere to documentation standards - To know ethical and legal issues - To undertake self-care |
| Cramer & Long (2018) | Not specified | To introduce and develop core competency skills in suicide prevention | - To recognise personal reasons, attitudes, and social norms concerning suicide - To identify evidence-based risk and protective factors, and theories of suicide - To understand the role of social support in suicide prevention - To articulate ethical and legal considerations concerning suicide - To describe public health approaches to suicide prevention - To understand vulnerable population needs in terms of suicide prevention - To use contemporary suicide prevention-related terminology - To describe mental health service approaches to suicide prevention |
| Cramer et al. (2016) | Suicide theory, risk assessment and management | To teach a streamlined set of core competencies in suicide risk assessment and managements as well as general learner objectives such as scholarly writing skills | - To know and manage your attitude and reactions toward suicide when with a client - To develop and maintain a collaborative, empathic stance toward the client, know and elicit evidence-based risk and protective factors - To focus on current plan and intent of suicidal ideation - To determine risk level - To develop and enact a collaborative evidence-based treatment plan while notifying and involving others - To document risk plan and clinical reasoning for decisions - To know the law concerning suicide - To engage in debriefing and self-care - To gain a broad-based command of psychological theory and literature on suicide - To develop specific expertise in one or more areas of suicide - To enhance critical thinking and analysis skills - To develop the ability to critically evaluate and apply research findings, and clinical and research writing skills |
| Desai et al, 2018 | Not specified | To improve the attitude of the trainee doctors toward suicide and equip them with necessary skills for communicating with depressed suicidal persons and offering the correct guidance and help to their peers and patients | Not specified |
| De Silva et al. (2015) | Suicide Awareness and Intervention Programme (SAIP) | To increase students’ knowledge and awareness about suicide-related issues, develop interpersonal skills around suicide screening and increase awareness of available support services | - To increase awareness and knowledge of suicide: risk factors, signs and indicators, diversity of pathways, protective factors - To develop skills and confidence in communicating with individuals who may be at risk of suicide - To apply a framework to assess an individual’s risk of suicide - Increase awareness and knowledge of local resources and support services to facilitate help seeking behaviour - To establish a network of individuals for personal and professional support - To reflect on personal motivation for involvement in the SAIP and limitations of role |
| El-Den et al. (2018) | Mental Health First Aid (MHFA) plus role play scenarios | Not specified | *General learning outcomes outlined on Mental Health First Aid website include:*   - To improve attitudes towards people with mental health difficulties - To reduce stigma - To improve knowledge and confidence in providing help and likelihood to advise people to seek professional help |
| Goh et al., 2016 | Mental Health Nursing Module | To provide nursing students with active participation in communication skills within a safe learning environment. | Not specified |
| Harshe et al, 2022 | Suicide sensitisation and prevention (SSP) workshop | Not specified | Not specified |
| Heyman et al, 2015 | Applied Suicide Intervention Skills Training (ASIST) | To recognize when someone may be at risk of suicide and work with them to create a plan that will support their immediate safety. | *General learning outcomes outlined on ASIST website include:*   - Understand the ways personal and societal attitudes affect views on suicide and interventions Provide guidance and suicide first-aid to a person at risk in ways that meet their individual safety needs - Identify the key elements of an effective suicide safety plan and the actions required to implement it Appreciate the value of improving and integrating suicide prevention resources in the community at large - Recognise other important aspects of suicide prevention including life-promotion and self-care |
| Hjelvik et al, 2022 | Talk Saves Lives | To provide participants with a clear understanding of suicide as a leading cause of death, including the most up-to-date research on suicide prevention, and what they can do in their communities to save lives. | - Restate the prevalence of mental illness and suicidal ideation among medical trainees - List modifiable and nonmodifiable risk factors and warning signs associated with suicide as a first step toward understanding how to provide resources to a peer in need - Describe the importance of asking directly about suicidal thoughts, intentions, and access to lethal means if a peer is showing warning signs - Demonstrate confidence in deploying skills to assist a suicidal peer - Explain how to direct a peer to routine care services, crisis care services, and local and national suicide prevention resources |
| Hill et al., 2024 | AS+K? About Suicide To Save A Life | To provide participants with basic suicide statistics and teach how to identify warning signs for suicide, how to ask about suicidal thoughts, and how to respond appropriately, including seeking emergency services. | Not specified |
| Hutson and Zeno, 2021 | Objective structured clinical examination (OSCE) - Clinical Competence for Youth Suicide | To build the requisite clinical skills for assessing and  managing paediatric patients in mental health crisis. | - Assess for suicidality in a child presenting with an acute mental health concern using an evidence-based screening tool - classify the severity of risk for suicide and select the appropriate disposition of care for a child with suicidal ideation - Formulate an individualized safety plan according to risk severity for a child with suicidal ideation and plan for follow up - Develop and document an appropriate management plan for a child with an acute mental health concern according to suicide risk severity |
| Jacobson et al., 2012 | Question, Persuade, and Referral (QPR) | Not specified | *General learning outcomes outlined on QPR website include:*   - Recognize the warning signs of suicide - Know how to offer hope - Know how to get help and save a life |
| Kerr et al. (2018) | SafeTALK | To help people recognise a person with suicidal thoughts and connect them to people who could carry out a full intervention. | - To recognise when someone is thinking about suicide - To connect them to help and support |
| Kourgiantakis et al., 2021 | Social Work Practice in Mental Health | Not specified | - Explain the role of social workers and their unique contributions in the field of mental health and addictions - Identify signs and symptoms of mental health and substance use concerns, as well as behavioural addictions - Assess mental health concerns, addictions, and suicide risk through role play and simulated assessments - Apply Motivational Interviewing skills in assessing mental health and addiction concerns - Develop a case formulation for simulated clients with mental health and/or addiction concerns; - Apply recovery-oriented, trauma-informed, and harm reduction models to mental health assessments - Apply principles of anti-oppressive practice and a culturally sensitive approach to mental health assessments - Increase critical self-awareness with respect to one’s own social location, experiences, values, assumptions, and biases related to mental health and addictions - Critically analyse the role of culture and its influence on service provisions, the helping relationship, and stigma in mental health and addictions |
| Kratz et al. (2020) | The Collaborative Assessment and Management of Suicidality (CAMS) framework | CAMS is a clinical philosophy that emphasizes collaboration and empathy for the patient’s suicidal state in the pursuit of suicide-specific interventions. | *General learning outcomes outlined on the Collaborative Assessment and Management website include:*   - To learn how to manage suicidal risk through collaboration - To understand various approaches to resolving situations with suicidal patients - To increase knowledge base and confidence in suicide prevention |
| Kullberg et al, 2020 | Professionals in Training to STOP (PITSTOP) suicide | Not specified | Not specified |
| LeCloux, 2021 | Assessment and Diagnosis | Not specified | Not specified |
| Lerchenfeldt et al, 2020 | Suicide Assessment and Management Team-Based Learning Module | Not specified | - Identify risk factors associated with suicide - Compare and contrast subgroups of the population that are at increased risk for suicide, and discuss how to eliminate disparities among these vulnerable groups - Indicate which medications are used to reduce the risk of suicide and which are used in the clinical management of suicidal individual - Discuss the key elements that must be evaluated in order to conduct a comprehensive assessment of a patient’s risk for suicide - Compare the major treatment strategies (acute and long-term) utilized to optimally treat patients who are assessed and believed to be at significant risk for suicide - Create a clinical management plan for a suicidal patient using evidence-based medicine - Discuss differences in state policies for efficient and effective continuity of care - Participate in the team-based learning activity in a professional and respectful manner - Engage the material by critically evaluating its content and employing peer teaching throughout the session |
| Lu et al, 2016 | Scenario-Based Learning on Suicidal Patient Care | Not specified | Not specified |
| Luebbert and Popkess, 2015 | Not specified | Not specified | Not specified |
| Magerman et al, 2022 ) | Not specified | Not specified | Not specified |
| McKeirnan et al., 2023 | Mental Health First Aid (MHFA) training | To teach participants how to communicate with and support people experiencing suicidal thoughts | General learning outcomes outlined on Mental Health First Aid website include:   - To improve attitudes towards people with mental health difficulties - To reduce stigma - To improve knowledge and confidence in providing help and likelihood to advise people to seek professional help |
| Mospan et al., 2017 | Communications Skills for Health Professionals - Addressing Feelings | To teach students how to respond and react to patient’s emotion. | Not specified |
| Muehlenkanp & Thoen (2019) | Understanding Suicide | Not specified | - To describe major theories of suicide - To identify the dominant risk and protective factors for suicide - To identify empirically supported approaches to suicide prevention and treatment - To understand the impact of suicide on individual, familial/ group, societal, and global levels - To explain ethical issues related to suicide and its research - To become familiar with special issues and controversies in the field of suicidology |
| Muehlenkamp and Quinn-Lee, 2023 | QPR | Not specified | *General learning outcomes outlined on QPR website include:*   - Recognize the warning signs of suicide - Know how to offer hope - Know how to get help and save a life |
| Nebhinani et al., 2020 | Not specified | Not specified | Not specified |
| Ng et al., 2022 | Not specified | To enable students to feel they can contribute to preventing suicide, and that they do not need to be experts to converse with others about suicide | Not specified |
| O'Reilly et al, 2019 | Mental Health First Aid (MHFA) training | To teach participants how to communicate with and support people experiencing suicidal thoughts | *General learning outcomes outlined on Mental Health First Aid website include:*   - To improve attitudes towards people with mental health difficulties - To reduce stigma - To improve knowledge and confidence in providing help and likelihood to advise people to seek professional help |
| Osteen, 2018 | QPR | Not specified | *General learning outcomes outlined on QPR website include:*   - Recognize the warning signs of suicide - Know how to offer hope - Know how to get help and save a life |
| Osteen et al., 2014 | QPR | Not specified | *General learning outcomes outlined on QPR website include:*   - Recognize the warning signs of suicide - Know how to offer hope - Know how to get help and save a life |
| Quemada-González et al., 2024 | N/A – content delivered as part of the Mental Health and Psychiatric II module | Not specified | Not specified |
| Patel et al, 2018 | Not specified | To identify signs  of depression and other behaviours that put individuals at heightened risk for suicide | Not specified |
| Perez et al., 2022 | Not specified | To develop skills prior to encountering difficult conversations in professional practice | Not specified |
| Phillips et al. (2019) | Not specified | To increase suicide awareness for medical students | - To increase their knowledge of the symptoms of depression and the risk factors for suicide - To provide a framework to discuss suicide - To encourage open discussion about suicide |
| Pothireddy et al., 2022 | Adapted Veteran Administration’s S.A.V.E. gatekeeper programme | Not specified | - Identify suicide warning signs exhibited by individuals in pharmacy settings - Describe how to appropriately refer an individual who is at risk of suicide - Practice in small groups how to respond to an individual who exhibits suicide warning signs |
| Price et al., 2022 | The Safety Planning Intervention | To improve students’ skill set and  the quality of care by ensuring that patients would discharge from the hospital with an SPI | Not specified |
| Pullen et al. (2016) | QPR | Not specified | - To learn how to Question, Persuade and Refer someone who may be suicidal - To learn how to get help for yourself or learn more about preventing suicide - To learn the common causes of suicidal behaviour - To learn the warning signs of suicide - To learn how to get help for someone in crisis |
| Ranahan, 2020 | Not specified | Not specified | Not specified |
| Retamero et al, 2014 | Not specified | Not specified | Not specified |
| Scott (2015) | Not specified | To introduce the fundamental knowledge and skills necessary for the understanding of and working practice with individuals at risk for suicidal thoughts, attempts, and completions | - To increase knowledge of suicide rates and statistics, warning signs, risk factors, and protective factors - To learn information on how to ask about suicide risk - To learn how to persuade at-risk individuals to seek help and referral resource information |
| Sharpe et al, 2014 | QPR | Not specified | *General learning outcomes outlined on QPR website include:*   - Recognize the warning signs of suicide - Know how to offer hope - Know how to get help and save a life |
| Stallman, 2020 | Care, Collaborate, Connect | To equip health professionals with the knowledge and skills to support all people when they are distressed, irrespective of what coping strategies they use. | Not specified |
| Takahashi et al., 2022 | Crisis-management, Anti-stigma, Mental health literacy Program for University Students (CAMPUS) | Not specified | Not specified |
| Vincent & David (2016) | Not specified | To teach pharmacy students how to prevent suicide | - To confront one’s values and emotions about the phenomenon of suicide - To understand the psychologic and existential dynamics of suicide - To evaluate the risk of suicide for an individual - To intervene appropriately with people having suicidal ideations - To be aware of local resources dedicated to mental health |
| Ward (2011) | SMILE: Simple, Mental Health Initiative in Education and Learning | To improve the knowledge and skills of third-year nursing students participating in their first clinical placement in mental healthcare | - To learn about how to reason through a problem - To discover problem-solving through self-directed learning - To apply knowledge of suicide and suicide prevention to the problem - To integrate knowledge and experience to solve the problem |
| Wathelet et al., 2023 | Mental health and suicide prevention training module | Not specified | Not specified |
| Willson et al. (2020) | Not specified | To increase student pharmacist knowledge and confidence regarding assessing and intervening (skills) with individuals considering suicide | - To explain suicide’s impact in communities - To demonstrate how a pharmacist can help prevent suicide - To integrate safer homes (safe storage and disposal) messaging into daily patient care - To apply LEARN (Look for warning signs, Empathise and Listen, Ask about suicide, Remove the danger, Next steps) |
| Witry et al. (2020) | QPR | Not specified | - To learn how to question, persuade and refer someone who may be suicidal - To learn how to get help for yourself or learn more about preventing suicide - To learn the common causes of suicidal behaviour - To learn the warning signs of suicide - To learn how to get help for someone in crisis |
| Witry et al. (2019) | Not specified | To provide first year PharmD students with foundational knowledge on suicide prevention | - To learn about suicide statistics and public health significance - To recognise protective factors, risk factors, and warning signs of suicidal ideation - To apply strategies for asking about suicidal ideation, including practice asking about suicide - To learn about resources and referral - To respond to brief case scenarios and questions by applying learned knowledge |
| Yousuf et al. (2013) | Not specified | The programme was designed to encourage students to adopt different attitudes towards suicide. | - To develop core competencies for mental health professionals dealing with suicidal patients identified by the American Association of Suicidology and the Suicide Prevention Resource Centre - To increase knowledge on suicide and suicidal behaviour, theories of suicide and deliberate self-harm, factors associated with suicide, suicide and ethics, stress, and mental health and well-being - To develop skills in risk assessment, prevention strategies, intervention strategies, postvention strategies - To learn about suicide among health professionals - To explore the concept of life, death, and the suicidal patient - To improve skills for overcoming stress |
